# Implementation of a Web-Based Resilience Enhancement Training for Nurses: Pilot Randomized Controlled Trial

**DOI:** 10.2196/43771

**Published:** 2023-02-14

**Authors:** Catherine Henshall, Zoe Davey, Cynthia Srikesavan, Liam Hart, Dan Butcher, Andrea Cipriani

**Affiliations:** 1 Oxford Institute for Nursing, Midwifery and Allied Health Research Oxford Brookes University Oxford United Kingdom; 2 Research and Development Oxford Health NHS Foundation Trust Oxford United Kingdom; 3 Department of Psychiatry University of Oxford Oxford United Kingdom

**Keywords:** burnout, COVID-19, health care setting, health care staff, health care provider, mental health, mental well-being, nurses, nursing, pilot trial, psychological health, resilience training, resilience, web-based health, web-based training

## Abstract

**Background:**

Global workforce challenges faced by health care providers are linked to low levels of job satisfaction, recruitment, retention, and well-being, with detrimental impacts on patient care outcomes. Resilience-building programs can provide support for staff who endure highly stressful environments, enhance resilience, and support recruitment and retention, with web-based formats being key to increasing accessibility.

**Objective:**

We aimed to examine participants’ engagement with a newly developed Resilience Enhancement Online Training for Nurses (REsOluTioN), explore its acceptability, and compare levels of resilience and psychological well-being in nurses who completed REsOluTioN with those who did not.

**Methods:**

We carried out a pilot randomized trial (1:1), conducted at a single site (mental health and community trust in South England) between August 2021 and May 2022. Local research ethics approvals were obtained. Nurses were invited to participate and were randomly assigned to a waitlist group or REsOluTioN group. Training lasted for 4 weeks, consisting of prereading, web-based facilitated sessions, and mentorship support. We evaluated trial engagement, acceptability of training, and pre-post changes in resilience, measured by the Brief Resilience Scale, and psychological well-being, measured by the Warwick Edinburgh Mental Wellbeing Scale. Qualitative participant feedback was collected. Consolidated Standards of Reporting Trials 2010 extension guidelines for reporting pilot and feasibility trials were used.

**Results:**

Of 108 participants recruited, 93 completed the study. Participants’ mean age was 44 (SD 10.85) years. Most participants were female (n=95, 88.8%), White (n=95, 88.8%), and worked in community settings (n=91, 85.0%). Sixteen facilitated and 150 mentoring sessions took place. Most REsOluTioN program participants reported the sessions helped improve their resilience (n=24, 72.8%), self-confidence (n=24, 72.7%), ability to provide good patient care (n=25, 75.8%), relationships with colleagues (n=24, 72.7%), and communication skills (n=25, 75.8%). No statistically significant differences between training and control groups and time on well-being (*F*_1,91_=1.44, *P*=.23, partial η^2^=0.02) and resilience scores (*F*_1,91_=0.33, *P*=.57, partial η^2^=0.004) were revealed; however, there were positive trends toward improvement in both. Nurse participants engaged with the REsOluTioN program and found it acceptable. Most found web-based training and mentoring useful and enjoyed learning, reflection, networking, and participatory sessions.

**Conclusions:**

The REsOluTioN program was acceptable, engaging, perceived as useful, and nurses were keen for it to be implemented to optimize resilience, psychological health, communication, and workplace environments. The study has evidenced that it is acceptable to implement web-based resilience programs with similar design features within busy health care settings, indicating a need for similar programs to be carefully evaluated. Mentorship support may also be a key in optimizing resilience. Trial limitations include small sample size and reduced statistical power; a multicenter randomized controlled trial could test effectiveness of the training on a larger scale.

**Trial Registration:**

ClinicalTrials.gov NCT05074563; https://clinicaltrials.gov/ct2/show/NCT05074563

**International Registered Report Identifier (IRRID):**

RR2-10.2196/37015

## Introduction

### Overview

The ongoing global workforce challenges facing health care providers are well documented and are linked to low levels of job satisfaction, recruitment, retention, and staff well-being, with detrimental impacts on patient care outcomes [1,2]. The central role of nurses in health care delivery is notable, as they are the largest group of health care professionals worldwide and are at the forefront of patient facing care [3]. However, the increased pressures they face in providing high-quality, complex patient care within overstretched and underresourced environments place many nurses under acute stress, testing their resilience [1,4-6]. In recent years, widespread media and public recognitions have been awarded to nurses who have continuously worked to protect patients, often to the detriment of their own health and well-being. The resulting physical, psychological, and emotional impact on nurses has been substantial, with an increase in mental health problems and burnout reported [7-9] and an increase in the number of nurses leaving the profession [10].

The development of evidence-based strategies to improve the psychological well-being of health care staff and mitigate against burnout has been cited as a key priority [11], with resilience-enhancement programs identified as one tool to help address this problem [12-15]. Resilience-building programs can provide targeted support for staff who are enduring unprecedented levels of stress and burnout, with recognized importance in contributing to increased psychological health and well-being in nurses [16-19]. They can also aid recruitment and retention within international health care organizations [20,21]. Resilience can be defined in many ways and is a dynamic rather than static construct, in which an individual’s resilience level can change depending on the fluctuating internal and external challenges they face [6,19]. In this paper, resilience is defined as an individual’s ability to “adjust to adversity, maintain equilibrium, retain some sense of control over their environment, and continue to move on in a positive manner” [19,22].

Several resilience-enhancement programs have been developed for health care professionals, using both group and individual programs, delivered in a range of contexts and using blended models of delivery [4,21,23-29]. These include resilience-building wellness apps [30], mentorship programs [20], Balint group sessions with colleagues, and access to resilience training programs for frontline health care staff [31]. Resilience programs have been found to enhance resilience and support retention and recruitment of health care staff [6,21,24-27,32], and systematic reviews have reported some evidence of their effectiveness and value in the health care population [22,24,33,34]. However, there is limited evidence regarding how these benefits are provided and which types of programs work for which staff and in what context. A recent review examining the use of web-based resilience-enhancement interventions showed them to have potential value in clinical practice settings by supporting staff who experienced prolonged workplace stress [35]. This is particularly important as the development of effective and evidence-based web-based programs can play a vital role in the current pandemic climate, where face-to-face training or meetings are often restricted or not allowed [36]. Web-based modes of delivery increase easy access and flexibility and decrease the need for face-to-face only interactions.

As a result of the need for targeted resilience-enhancement programs within the nursing setting, this paper reports on a pilot randomized controlled trial (RCT) that was designed to evaluate a web-based resilience-enhancement program for nurses [37]. The web-based training program, Resilience Enhancement Online Training for Nurses (REsOluTioN), was based on a face-to-face resilience-enhancement training program that had been previously piloted with nurses [6,29]. The design of the training program was also informed by a systematic review examining the effectiveness of web-based interventions to enhance resilience in health care professionals [35] and focus groups with nurses to gather information on what they felt should be the key features of such training programs [38]. During the focus group discussions, nurses viewed web-based programs aimed at enhancing resilience positively, highlighting potential benefits around combating burnout, managing stress, regaining work-life balance, mentorship, and building networks of support. Nurses emphasized the importance of branding, organizational support, and time in maximizing engagement with such a program.

### Study Aim

The aim of this paper is to report on the implementation and evaluation of the REsOluTioN pilot RCT. Specific study objectives were to (1) explore participants’ engagement with the REsOluTioN trial, assessed by the number of nurses recruited to it; (2) explore the acceptability of the REsOluTioN program, assessed by participant retention numbers and data on how the training impacted participants’ views on resilience, communication, clinical practice, and workplace relationships; and (3) compare levels of resilience and psychological well-being in nurses who completed REsOluTioN program with nurses who did not (waitlist control arm).

## Methods

### Trial Design and Setting

The study was a 1:1 two-armed pilot randomized trial. It was conducted in a mental health and community National Health Service (NHS) trust in the South of England.

### Ethical Considerations

The Oxford Brookes University Faculty Research Ethics Committee (F.20.01.12.1, dated August 22, 2021) reviewed and approved the trial protocol. Other necessary local research and development office approvals were obtained from the Oxford Health NHS Foundation Trust Research and Development Department (21/HRA/1418). The trial protocol was registered on ClinicalTrials.gov (NCT05074563). Consolidated Standards of Reporting Trials (CONSORT) 2010 extension guidelines for pilot and feasibility trials were used [39]. Web-based informed consent was obtained from all participants prior to study participation. All study data were deidentified to protect the privacy and confidentiality of participants. No financial incentives were provided; however, participants who completed the study did receive a continuous professional development certificate.

### Recruitment

We invited nonagency nurses of different levels of seniority, working across a wide range of clinical settings from the participating NHS trust to participate. We used posters on the participating trust’s website, social media platforms, and meetings with nursing staff, research delivery teams, and trust communications teams to promote the study. The participating trust’s chief nurse also shared information about the trial with all nurses employed by the trust, using email communications and web-based meeting forums. Nurses who were interested in taking part were provided with a Qualtrics survey web link that contained a study information sheet and consent form. Upon providing consent, they were asked to provide demographic information and complete a prestudy survey (Multimedia Appendix 1).

### The REsOluTioN Program

The REsOluTioN program was hosted on the Totara learning management system (version 12), via the Learning and Development information technology team at the participating trust. The web-based training was conducted over 4 weeks and covered weekly modules on (1) building hardiness and maintaining a positive outlook, (2) intellectual flexibility and emotional intelligence, (3) reflective and critical thinking, and (4) achieving life balance and enabling spirituality.

A blended synchronous and asynchronous learning approach was used, which included (1) web-based 4×120 minutes large-group facilitated sessions on the weekly modules led by experienced senior nurses and other senior multidisciplinary health care staff; (2) 4×30 minute independent preparatory learning on the module topics prior to the large-group facilitated sessions; and (3) 8 small group mentoring sessions led by senior nurses and delivered between 30 and 60 minutes at flexible timings, twice weekly.

Both large-group facilitated sessions and mentor meetings were delivered via Teams (Microsoft Corp). Learning materials in the form of PowerPoint presentations, instructional videos created by the study team, case examples, and peer-reviewed journal articles were used. The facilitated sessions also included group discussions and breakout activities. More about the content and delivery of REsOluTioN program is found elsewhere [37]. To gain a training completion certificate, participants randomized to the REsOluTioN program were expected to attend all the large-group facilitated sessions and at least one mentor meeting each week.

### Waitlist Control

Nurses who were randomized to the control arm were allocated to a waitlist for 6 weeks. After 6 weeks, participants from both arms were asked to complete a poststudy survey (Multimedia Appendix 2).

### Outcomes

The following outcome data were collected: (1) *participant engagement*: data were collected on the number of nurses who expressed an interest in joining the trial and those randomized to assess recruitment success and participant engagement with the trial. (2) *Acceptability of the REsOluTioN program*: data were collected on how many participants were retained in the REsOluTioN program. Baseline Likert-style data were collected from all participants via a web-based prestudy survey to examine their understanding of resilience and the anticipated usefulness of the REsOluTioN tool in enhancing resilience levels, confidence, views on clinical practice, communication skills, and relationships with workplace colleagues. Six weeks later, participants were provided with this information again in a web-based poststudy survey to monitor any changes over time. (3) *Resilience and Psychological Well-being*: the validated Brief Resilience Scale [40] and Warwick-Edinburgh Mental Wellbeing Scale [41] were used to measure changes in resilience, psychological health, and well-being over time between the REsOluTioN program and the waitlist control arms at 6 weeks.

### Data Management

All data were deidentified using study codes and stored in password-protected Excel (Microsoft Corp) spreadsheets that were accessed by authorized team members only. We followed the university’s policies and General Data Protection Regulation requirements for data storage.

### Sample Size

The limitations of the pilot study design, as well as finite resource availability, determined our sample size. The study objective was to afford a preliminary comparison of training outcomes, and due to funding constraints and the pressures imposed by COVID-19, we aimed to recruit between 60 and 100 participants; this was deemed appropriate for a pilot study of this nature [42]. We aimed to recruit participants over 4 cohorts to maximize recruitment potential due to the study being conducted during the pandemic and nurse participants having very busy work schedules. As a result, having a choice of 4 cohorts aided flexibility in terms of nurse participants’ being able to take part in the study alongside their work schedules. Though we intended for approximately 25 participants to be enrolled per cohort, there was variation in cohort size; this is reflective of nurse participants’ varying levels of availability throughout the study.

### Randomization and Blinding

An independent team member who was not involved in the conduct of the trial, delivery of the REsOluTioN program, or data analysis implemented the randomization and allocation. For randomization, we used a computer-generated random number sequence. For allocation concealment, we used sequentially numbered opaque-sealed envelopes that were opened only after entering the name of each participant on the envelope. A team member who was involved in data analysis was blinded to the group allocation. However, participants were not blinded to group allocation due to the nature of the training.

### Statistical Methods

Participants’ demographic characteristics and acceptability outcomes (completers or noncompleters) were descriptively analyzed. Depending on the normality of the data, resilience and psychological well-being measures were presented as means (SD) or medians (IQR). Intention-to-treat analysis was carried out to examine outcomes. Data from participants who withdrew from the study and who were lost to follow up (n=14, 13.1%) were imputed using the expectation maximization technique. We used ANOVA to evaluate any differences in resilience and psychological well-being between arms at 6 weeks. All analyses were undertaken in SPSS statistics software (version 22.0; IBM Corp) [43], with a significance level set at 0.05.

## Results

### Overview

**Figure 1:**
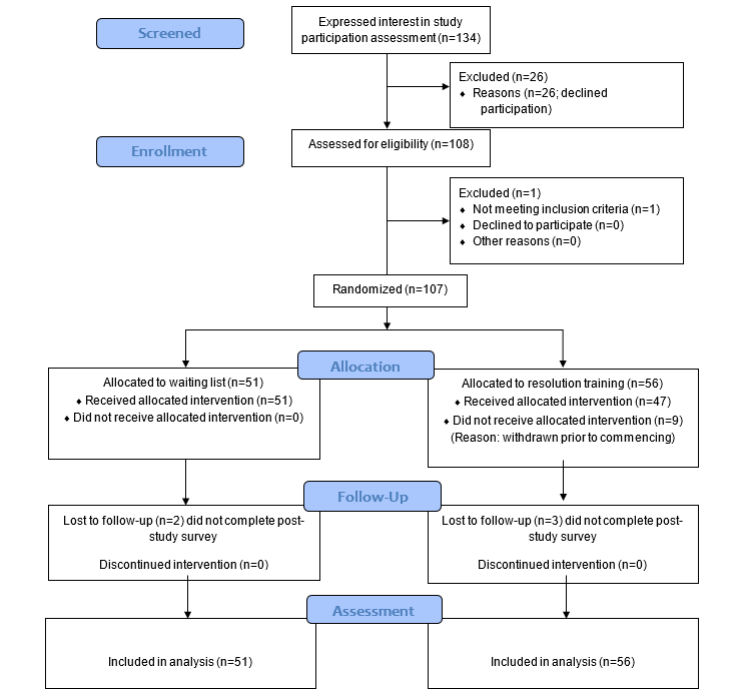
Consolidated Standards of Reporting Trials (CONSORT) flow diagram depicting flow of participants through the pilot trial.

A CONSORT diagram detailing the flow of participants through the pilot trial is presented in Figure 1.

### Characteristics of Trial Participants

Baseline characteristics of trial participants are presented in Table 1. The mean age of participants was 44 (SD 10.85) years; ages ranged between 24 and 69 years. Most participants were female (n=95, 88.8%), White (n=95, 88.8%), and working in the community setting (n=91, 85.0%). Participants were from a range of NHS clinical bands (band 4-8), with band 6 being the most common (n=47, 43.9%). A small number (n=5) of band 4 nursing associates were included, as they were experienced members of staff embedded within clinical teams during the pandemic, working alongside registered nurses in extended roles. The mean number of years of experience working in the nursing profession was 15.75 (SD 11.58).

Participants randomized to the REsOluTioN program (n=56) did not differ significantly from those randomized to the control group (n=51) with regard to age (t_105_=-0.26, *P*=.80), years working in the profession (t_105_=0.82, *P*=.42), gender (*χ*^2^_1_=0.2, *P*=.66), ethnicity (*χ*^2^_1_=1.96, *P*=.16), banding (*χ*^2^_1_=0.3, *P*=.57), nursing field (*χ*^2^_1_=0.8, *P*=.38), well-being (t_105_=0.56, *P*=.58)-, or resilience (t_105_=0.58, *P*=.56) outcomes at baseline (Table 1). The baseline, prestudy survey indicated that all participants (n=107, 100%) recognized the importance of personal resilience in the workplace, with the majority rating it as extremely important (n=88, 82.2%).

**Table 1:** Baseline characteristics of participants, mean psychological well-being, and resilience at baseline and 6 weeks.

Characteristics			Total	REsOluTioN^a^ group	Waitlist control group
Number of participants randomized, n			107	56	51
Age in years, mean (SD)			43.78 (10.85)	44.04 (10.72)	43.49 (11.08)
**Gender, n (%)**					
	Male	12 (11.2)		7 (12.5)	5 (9.8)
	Female	95 (88.8)		49 (87.5)	46 (90.2)
**Ethnicity, n (%)**					
	White	95 (88.8)		52 (92.9)	43 (84.3)
	Asian	3 (2.8)		0 (0.0)	3 (5.9)
	Black	7 (6.5)		2 (3.6)	5 (9.8)
	Mixed	2 (1.9)		2 (3.6)	0 (0.0)
**Work experience in years, mean (SD)**			15.75 (11.58)	14.88 (11.28)	16.71 (11.94)
**Level of NHS^b^ bands, n (%)**					
	Band 4	5 (4.7)		4 (7.1)	1 (2.0)
	Band 5	16 (15.0)		7 (12.5)	9 (17.6)
	Band 6	47 (43.9)		26 (46.4)	21 (41.2)
	Band 7	21 (19.6)		11 (19.6)	10 (19.6)
	Band 8 and above	18 (16.8)		8 (16.8)	10 (19.6)
**Type of work setting, n (%)**					
	Community and mental health services	91 (85.0)		46 (82.1)	45 (88.2)
	Forensics	4 (3.7)		3 (5.4)	1 (2.0)
	Corporate	6 (5.6)		2 (3.6)	4 (7.8)
	Learning disabilities	6 (5.6)		5 (8.9)	1 (2.0)
**Resilience, mean (SD)**					
	Baseline	3.02 (0.27)		3.01 (0.25)	3.04 (0.28)
	6 weeks	3.02 (0.26)		3.02 (0.25)	3.02 (0.26)
**Psychological well-being, mean (SD)**					
	Baseline	46.35 (7.52)		46.73 (8.32)	45.92 (6.59)
	6 weeks	47.56 (9.26)		48.55 (9.64)	46.47 (8.78)

^a^REsOluTioN: Resilience Enhancement Online Training for Nurses.

^b^NHS: National Health Service.

### Participant Engagement and Retention

Between August 2021 and May 2022, a total of 134 nurses expressed an interest in participating in the study. Of 134, a total of 108 completed the web-based consent process and prestudy survey. One participant was excluded on the basis of eligibility as he/she was a nursing student rather than an employed member of staff. Consented participants (n=107) were randomly assigned to the waitlist control (n=51) or REsOluTioN program (n=56). Nine participants withdrew from the REsOluTioN group prior to the intervention starting due to changing work commitments or annual leave requirements, which meant that they were unable to be allocated to a training cohort. At 6 weeks post enrollment, 93 participants had completed the poststudy survey, as 5 were lost to follow up and did not complete (2 in the waiting list group and 3 in the REsOluTioN group). When comparing participants who completed the study with those who withdrew or were lost to follow-up, there were no significant differences in terms of baseline demographic characteristics age (t_105_=0.92, *P*=.36), years working in the profession (t_105_=0.12, *P*=.99), gender (*P=*.51), ethnicity *(P=*.49), banding (*χ*^2^_1_=0.004, *P*=.95), nursing field (*P=*.65), resilience (t_105_=1.25, *P*=.22), or psychological well-being (t_105_=1.23, *P*=.22).

### Acceptability of the REsOluTioN Program

Participants were allocated to waitlist control and REsOluTioN groups in 4 consecutive cohorts during the recruitment period. Cohort 1 had 14 people in the waitlist control and 6 in the REsOluTioN group, cohort 2 had 2 and 4, cohort 3 had 13 and 11, and cohort 4 had 22 and 26 participants, respectively. A total of 150 mentoring sessions and 16 facilitated sessions took place across the 4 cohorts. No mentors or facilitators reported the length of the sessions, but the mentoring sessions lasted between 30 minutes to 1 hour depending upon the number of participants allocated to the mentor, while the large-group facilitated sessions lasted up to 2 hours. Thus, the sessions were acceptable in terms of delivery and duration. The participant retention rate at 6 weeks was 96% (44/47) in the REsOluTioN group; of these, 33 (75%) participants provided extra feedback on the REsOluTioN program via an additional survey.

Of the participants who completed the poststudy evaluation of the REsOluTioN program (n=33), the majority thought that both the web-based workplace resilience training (n=30, 90.9%) and mentoring (n=29, 87.9%) had been useful; a minority felt that their participation in the training program had not impacted on their experience or outlook toward clinical practice (n=7, 21.2%). Participants felt that participation in the web-based training had been important for improving their levels of resilience (n=24, 72.8%), self-confidence (n=24, 72.7%), belief in their ability to provide good patient care (n=25, 75.8%), relationships with work colleagues (n=24, 72.7%), and communication skills with colleagues (n=25, 75.8%).

Most participants who completed poststudy evaluation also found the content and sessions helpful (n=29, 87.9%) thought the training delivered an appropriate amount of information (n=27, 81.8%) and that the 4-week duration was about right for training of this type (n=23, 69.7%). The sessions on emotional intelligence and intellectual flexibility were rated most favorably, with 75.8% (n=25) of them indicating that they found it particularly helpful.

Analysis of free-text responses from REsOluTioN program participants suggested that they enjoyed opportunities for learning and reflection offered by the training as well as the chance to build networks and interact with colleagues at all levels. Participants indicated that they enjoyed the networking, mentorship, and participatory sessions the most (n=16, 51.5%). However, ringfencing time to attend and engage with the sessions was the most reported challenge due to pressurized working environments. One participant highlighted the need for a cultural shift to support people to take more time for their own well-being. Participants also highlighted organization-wide issues and variability in the way sessions were run as detracting from their overall experience. Table 2 provides a summary of illustrative quotes.

**Table 2:** Evaluation of Resilience Enhancement Online Training for Nurses (REsOluTioN) program–Illustrative quotes.

Theme	Illustrative quotes
Opportunities for learning and reflection	“*I feel I have learned something about myself and am very grateful to have attended this*.” [Band 6, Female, Community and Mental Health]
Building networks and interacting with colleagues	“*I enjoyed having senior managers actively taking part in the interactive discussions and group work, because it made me feel connected and listened to*.” [Band 6, Female, Community and Mental Health] “*[I enjoyed that] participants included a good range of nurses from very different professional backgrounds and specialities*.” [Band 5, Female, Community and Mental Health]
Networking, mentorship, and participatory sessions	“*They were all interesting sessions and it was good to have the protected time to review these subjects and reflect on my own feelings and development*.” [Band 7, Female, Community and Mental Health] “*The mentor sessions were the most rewarding – quickly building rapport with a small supportive group, where you can cry, laugh or vent*.” [Band 7, Female, Community and Mental Health]
Time as a barrier	“*It was sometimes difficult to attend the whole session or focus on it completely due to the responsibilities of my role. I tried to protect the time but this was not always possible which was frustrating at times*.” [Band 7, Female, Community and Mental Health]
Culture as a barrier	“*I feel a longer term…approach…would be really helpful and cement the belief that you deserve time to think about yourself regularly, to carve out headspace to consider yourself before reaching crisis*.” [Band 7, Female, Community and Mental Health]
Better organization of the sessions	“*I think it would have been useful to set out some ground rules*.” [Band 6, Male, Community and Mental Health] “*It would have been useful to have the pre-reading. I never received any information before the sessions*.” [Band 5, Female, Community and Mental Health]

### Resilience and Psychological Well-being

Mean resilience and psychological well-being scores at baseline and 6 weeks for both groups are presented in Table 1. There were very little pre-post differences in resilience scores across control group (mean 0.01, SD 0.35) and REsOluTioN group (mean 0.01, SD 0.27). The mean pre-post difference in psychological well-being scores was smaller in the control group (mean 0.55, SD 6.77) than in the REsOluTioN group (mean 1.82, SD 6.53).

Two-way mixed ANOVAs revealed no statistically significant differences between groups and time (baseline and 6 weeks) on resilience scores (*F*_1,105_=0.20, *P*=.66, partial η^2^=0.002) and well-being scores (*F*_1,105_=0.97, *P*=.33, partial η^2^=0.09).

## Discussion

### Principal Findings

Our findings have shown that nurse participants engaged with REsOluTioN program, as evidenced by the high recruitment rate to the pilot study. The training was acceptable to nurses working in frontline clinical settings; this is demonstrated by the large number of participants who enrolled in and completed the study. While a small number of participants withdrew from the study or were lost to follow-up, the majority of these withdrew prior to the training commencing due to changing work commitments, meaning that they were no longer able to join a cohort. Participant feedback demonstrated that most participants clearly valued the importance of protecting their resilience within the workplace. Most participants were receptive toward the REsOluTioN program and reported that they felt it was an important tool for helping to enhance their resilience, confidence, patient care provision, and relationships with work colleagues; this demonstrates the potential application of web-based resilience tools for this population group. Overall, the REsOluTioN program was well received and identified a desire from nurses for web-based resilience training tools to be implemented as a way of optimizing resilience, psychological health, communication practices, and the workplace environment. Though there were no significant differences between REsOluTioN and waitlist control groups at 6 weeks in terms of resilience and psychological well-being, these results must be considered alongside the small sample size, due to the pilot nature of the trial.

### Comparison With Prior Work

Our findings have highlighted the potential need for resilience-enhancement programs for nurses working in highly stressful working conditions. This pilot trial was carried out across an NHS trust in South England during the height of the COVID-19 pandemic, when pressures on health care staff were extremely high, with nurses having considerable demands on their time and multiple conflicting priorities [1,4,5,23,44]. Despite this, we managed to recruit on time and reach our target, implement the REsOluTioN program successfully, and collect data at the relevant time points. Hence, our study has provided initial evidence that it is acceptable to implement web-based resilience-enhancement programs with similar design features within busy health care settings; this has been identified in the wider literature prior to the pandemic [35], but to our knowledge has not been tested since COVID-19. Thus, these findings will enable larger scale studies to test the effectiveness of web-based resilience programs for nurses in terms of increasing resilience and psychological well-being.

The REsOluTioN pilot trial has shown that web-based resilience-enhancement programs appear acceptable to nurses working across a range of health care settings. This is evidenced by the low dropout rate of participants who enrolled in the study, which is an indicator of high levels of acceptance [45]. This was further evidenced by the qualitative feedback from participants who enrolled in the training arm of the study. These findings provide evidence of nurses’ engagement and satisfaction with the program. This indicates a real need for carefully evaluated web-based resilience-enhancement programs for nurses to be implemented across health care settings.

Feedback from participants who enrolled in the training arm indicated that they were satisfied with the different the REsOluTioN program components and that the web-based facilitated and mentorship sessions were well received. This endorsement of the mentorship component of the REsOluTioN program has been evidenced in previous research exploring resilience in nurses [6,25,29], suggesting that mentorship support may be a key component in optimizing resilience among the nursing workforce. Future studies could focus on evaluating mentorship-based sessions for nurses, as these have the potential to be flexibly implemented and tailored to the needs of individuals working across a range of health and social care settings. Furthermore, focus groups carried out with nurses in the participating trust indicated a need for resilience training, with nurses identifying that supportive measures to help combat workplace stressors and burnout were acutely needed [38]. This suggests that the design features, content, and format of REsOluTioN are appropriate and acceptable for use in this population and could be replicated and adapted for use in larger populations using a large-scale RCT design.

Although no statistically significant mean differences were found in levels of resilience or psychological well-being between the waitlist control and training arms, the small sample size of the pilot study makes it difficult to draw meaningful conclusions from the dataset. Furthermore, resilience and psychological well-being scores were higher in the training arm than the waitlist control arm at the end of the study, suggesting positive trends in the right direction and that the REsOluTioN program may lead to statistically significant outcomes when tested on a larger population. Pilot studies are recommended as the first step in determining information relating to study design, engagement, and acceptability, as *P* values alone can produce arbitrary results that can lead to potentially important findings being missed [46]. A fully powered, multicenter RCT is required to test the effectiveness of the REsOluTioN program on resilience and psychological well-being scores in a larger population.

### Strengths and Limitations of the Study

To our knowledge, REsOluTioN was the first web-based training piloted on nurses working during the COVID-19 pandemic and has highlighted many benefits of providing such a resource to nurses working under highly pressurized conditions. In addition, the engagement and acceptability outcomes have been achieved as indicated by the high study recruitment and adherence rates and from the qualitative survey feedback. This confirms that it is possible to implement web-based resilience training programs for nurses within busy workplace environments with successful engagement from nurses.

Study limitations include the small sample size, and hence robust conclusions on psychological well-being and resilience outcomes cannot be made. In addition, this study was conducted at only one NHS trust; future studies should be conducted across a range of NHS health and social care settings to increase the generalizability of the findings. Finally, conducting a study during the COVID-19 pandemic may have influenced the findings, as nurses were working under even more pressurized conditions than usual [1,23,44], which may have influenced their responses due to heightened levels of stress and anxiety. Care must be taken to be aware of these issues when implementing similar resources across health and social care settings in the peri- and postpandemic era.

### Conclusions

This pilot RCT has identified the importance of, and need for, tailored resilience-enhancement programs for nurses, who are facing unprecedented workforce pressures and may benefit from additional forms of structured support. The components of the REsOluTioN program were well received, with specific emphasis placed on the mentorship components, suggesting this may be an area of future research focus and practical application.

Further, large-scale research is required to test the effectiveness of the REsOluTioN program across real-world health care settings, with the aim of increasing the sustainability, health, and well-being of the nursing workforce in the future.

## Appendix

Multimedia Appendix 1 Preintervention Survey.

Multimedia Appendix 2 Postintervention Survey and Evaluation.

Multimedia Appendix 3 CONSORT-eHEALTH checklist (V 1.6.2).

## Abbreviations

CONSORT: Consolidated Standards of Reporting Trials
NHS: National Health Service
RCT: randomized controlled trial
REsOluTioN: Resilience Enhancement Online Training for Nurses

## Data Availability

The data that support the findings of this study are available from the corresponding author upon reasonable request.
