# Supplementary material for: Implementation of a Web-Based Resilience Enhancement Training for Nurses: Pilot Randomized Controlled Trial
Source: J Med Internet Res. 2023 Feb 14;25:e43771. doi: 10.2196/43771 (PMC9975925; doi:10.2196/43771)
Supplement: Multimedia Appendix 1 [file jmir_v25i1e43771_app1.docx]

**Multimedia Appendix 1**

**Preintervention Survey**

| **Question** | **Response** |
| --- | --- |
| What is your age in years? |  |
| What best describes your gender? | Male  Female  Prefer to self-describe |
| Which band are you? | Band 5  Band 6  Band 7  Band 8a/8b/8c/8d  Other |
| How many years have you been working in your profession? |  |
| What clinical setting do you work in? | Forensics  Community  Learning disabilities  Corporate |
| What is your ethnicity? | White: British/Other white groups  Black: British/African/ Caribbean/Other black groups Asian: British/Indian/ Pakistani/ Bangladeshi/other Asian groups Chinese: British/Other Chinese groups Other Mixed |
| This online tool aims to improve your personal resilience and wellbeing in the workplace. How useful do you feel this will be?  You will work with a mentor via the tool. How useful do you feel this will be? | Not useful at all  Not very useful  Moderately useful  Largely useful  Extremely useful |
| How important do you feel it is to have personal resilience in your workplace? | Not important at all  Not very important  Moderately important  Largely important  Extremely important |
| Below are some statements about resilience. Please select that answer that best describes you [40]:  I tend to bounce back quickly after hard times  I have a hard time making it through stressful events  It does not take me long to recover from a stressful event  It is hard for me to snap back when something bad happens  I usually come through difficult times with little trouble  I tend to take a long time to get over set-backs in my life | Strongly disagree  Disagree  Neutral  Agree  Strongly agree |
| Below are some statements about feelings and thoughts. Please tick the box that best describes your experiences of each over the last **2** weeks [41]:  I’ve been feeling optimistic about the future  I’ve been feeling useful  I’ve been feeling relaxed  I’ve been feeling interested in other people  I’ve had energy to spare  I've been dealing with problems well  I've been thinking clearly  I've been feeling good about myself  I've been feeling close to other people  I've been feeling confident  I've been able to make up my own mind about things  I've been feeling loved  I've been interested in new things  I've been feeling cheerful | None of the time  Rarely  Some of the time  Often  All of the time |
| How important do you feel this online tool could be for improving your own:  Level of personal resilience?  Level of self-confidence in the workplace?  Belief in your ability to provide good patient care?  Relationship with your work colleagues?  Communication skills with your colleagues? | Not important at all  Not very important  Moderately important  Largely important  Extremely important |
| Do you think four weeks is too long too short or about right? | Too long  Long  About right  Short  Too short |
| Do you feel that an online tool with a resilience component could impact on your experience or outlook towards clinical practice? | Yes  Maybe  No |
