# Supplementary material for: Implementation of a Web-Based Resilience Enhancement Training for Nurses: Pilot Randomized Controlled Trial
Source: J Med Internet Res. 2023 Feb 14;25:e43771. doi: 10.2196/43771 (PMC9975925; doi:10.2196/43771)
Supplement: Multimedia Appendix 2 [file jmir_v25i1e43771_app2.docx]

**Multimedia Appendix 2**

**Postintervention Survey and Evaluation**

| **Question** | **Response** |
| --- | --- |
| Below are some statements about resilience. Please select that answer that best describes you [40]:  I tend to bounce back quickly after hard times  I have a hard time making it through stressful events  It does not take me long to recover from a stressful event  It is hard for me to snap back when something bad happens  I usually come through difficult times with little trouble  I tend to take a long time to get over set-backs in my life | Strongly disagree  Disagree  Neutral  Agree  Strongly agree |
| Below are some statements about feelings and thoughts. Please tick the box that best describes your experiences of each over the last **2** weeks [41]:  I’ve been feeling optimistic about the future  I’ve been feeling useful  I’ve been feeling relaxed  I’ve been feeling interested in other people  I’ve had energy to spare  I've been dealing with problems well  I've been thinking clearly  I've been feeling good about myself  I've been feeling close to other people  I've been feeling confident  I've been able to make up my own mind about things  I've been feeling loved  I've been interested in new things  I've been feeling cheerful | None of the time  Rarely  Some of the time  Often  All of the time |
| **Completed by Intervention Group Only** |  |
| How useful have you found the online training? | Not useful at all  Not very useful  Moderately useful  Largely useful  Extremely useful |
| Was an appropriate amount of information given via the training? | Not enough information Appropriate amount of information  Too much information |
| How useful has it been to have a mentor? | Not useful at all  Not very useful  Moderately useful  Largely useful  Extremely useful |
| How likely are you to keep in touch with your mentor after this programme has ended? | Definitely won't  Not very likely  Not sure Very likely  Definitely |
| Was there a session you found particularly helpful, and if so, why? | Building hardiness and maintaining a positive outlook  Emotional intelligence and intellectual flexibility Reflective and critical thinking Spirituality and achieving life balance  All were equally helpful  None were helpful  Additional comments: |
| Can you tell us what you enjoyed most about the online training? | Functionality and accessibility of the platform  Mentorship Facilitated sessions Content including pre-work  Duration Networking and participatory sessions  Time and space away from work  Other  Additional comments: |
| How important do you feel this online tool has been for improving your own:  Level of personal resilience?  Level of self-confidence in the workplace?  Belief in your ability to provide good patient care?  Relationship with your work colleagues?  Communication skills with your colleagues? | Not important at all  Not very important  Moderately important  Largely important  Extremely important |
| Do you think four weeks has been too long too short or about right? | Too long  Long  About right  Short  Too short |
| Do you feel that your experience or outlook towards clinical practice has changed as a result of the programme? | Yes  Maybe  No |
