# Supplementary material for: Implementation of a Web-Based Resilience Enhancement Training for Nurses: Pilot Randomized Controlled Trial
Source: J Med Internet Res. 2023 Feb 14;25:e43771. doi: 10.2196/43771 (PMC9975925; doi:10.2196/43771)
Supplement: Multimedia Appendix 3 [file jmir_v25i1e43771_app3.pdf]

CONSORT-EHEALTH Checklist V1.6.2 Report

Manuscript Number: 43771

(based on CONSORT-EHEALTH V1.6), available at [http://tinyurl.com/consort-ehealth-v1-6].

Date completed

2/11/2023 12:09:14

by

Catherine Henshall

Implementation of a Web-Based Resilience Enhancement Training for Nurses: A Pilot Randomized Controlled Trial

TITLE

1a-i) Identify the mode of delivery in the title

"Implementation of a Web-Based Resilience Enhancement Training for Nurse s: Pilot Randomized Controlled Trial"

1a-ii) Non-web-based components or important co-interventions in title

there are no additional components

1a-iii) Primary condition or target group in the title

"Implementation of a Web-Based Resilience Enhancement Training for Nurses: Pilot Randomized Controlled Trial"

ABSTRACT

1b-i) Key features/functionalities/components of the intervention and comparator in the METHODS section of the ABSTRACT

"Nurses   were invited to participate and were randomly assigned to a waitlist group or REsOluTioN group. Training lasted for 4 weeks, consisting of pre-reading, web- based-facilitated sessions, and mentorship support. "

1b-ii) Level of human involvement in the METHODS section of the ABSTRACT

not applicable to the paper

1b-iii) Open vs. closed, web-based (self-assessment) vs. face-to-face assessments in the METHODS section of the ABSTRACT

information inlcuded in the paper

1b-iv) RESULTS section in abstract must contain use data

yes, this information is in the paper

1b-v) CONCLUSIONS/DISCUSSION in abstract for negative trials

Information available in accepted paper

INTRODUCTION

2a-i) Problem and the type of system/solution

"The development of evidence-based strategies to improve the psychological well-being of health care staff and mitigate against burnout has been cited as a key priority [11], with resilience-enhancement programs identified as one tool to help address this problem [12-15]. Resilience-building programs can provide targeted support for staff who are enduring unprecedented levels of stress and burnout, with recognized importance in contributing to increased psychological health and well-being in nurses [16-19]. They can also aid recruitment and retention within international health care organizations [20,21].... As a result of the need for targeted resilience-enhancement programs within the nursing setting, this paper reports on a pilot randomized controlled trial (RCT) that was designed to evaluate a web-based resilience-enhancement program for nurses [37]. The web-based training program, Resilience Enhancement Online Training for Nurses (REsOluTioN), was based on a face-to-face resilience-enhancement training program that had been previously piloted with nurses [6,29]. The design of the training program was also informed by a systematic review examining the effectiveness of web-based interventions to enhance resilience in health care professionals [35] and focus groups with nurses to gather information on what they felt should be the key features of such training programs [38]. "

2a-ii) Scientific background, rationale: What is known about the (type of) system

"Several resilience-enhancement programs have been developed for health care professionals, using both group and individual programs, delivered in a range of contexts and using blended models of delivery [4, 21,23-29]. These include resilience-building wellness apps [30], mentorship programs [20], Balint group sessions with colleagues, and access to resilience training programs for frontline health care staff [31]. Resilience programs have been found to enhance resilience and support retention and recruitment of health care staff [6,21,24-27,32], and systematic reviews have reported some evidence of their effectiveness and value in the health care population [22,24,33,34]. However, there is limited evidence regarding how these benefits are provided and which types of programs work for which staff and in what context. A recent review examining the use of web-based resilience-enhancement interventions showed them to have potential value in clinical practice settings by supporting staff who experienced prolonged workplace stress [35]. This is particularly important as the development of effective and evidence-based web-based programs can play a vital role in the current pandemic climate, where face-to-face training or meetings are often restricted or not allowed [36]. Web-based modes of delivery increase easy access and flexibility and decrease the need for face-to-face only interactions. "

Does your paper address CONSORT subitem 2b?

"The aim of this paper is to report on the implementation and evaluation of the REsOluTioN pilot RCT. Specific study objectives were to
(1) explore participants' engagement with the REsOluTioN trial, assessed by the number of nurses recruited to it;
(2) explore the acceptability of the REsOluTioN program, assessed by participant retention numbers and data on how the training impacted participants' views on resilience, communication, clinical practice, and workplace relationships; and(3) compare levels of resilience and psychological well-being in nurses who completed REsOluTioN program with nurses who did not (waitlist control arm)."

METHODS

3a) CONSORT: Description of trial design (such as parallel, factorial) including allocation ratio

"The study was a 1:1 two-armed pilot randomized trial. "

3b) CONSORT: Important changes to methods after trial commencement (such as eligibility criteria), with reasons

Not applicable for this study

3b-i) Bug fixes, Downtimes, Content Changes

4a) CONSORT: Eligibility criteria for participants

"We invited nonagency nurses of different levels of seniority, working across a wide range of clinical settings from the participating NHS trust to participate. "

4a-i) Computer / Internet literacy

4a-ii) Open vs. closed, web-based vs. face-to-face assessments:

"We used posters on the participating trust's website, social media platforms, and meetings with nursing staff, research delivery teams, and trust communications teams to promote the study. The participating trust's chief nurse also shared information about the trial with all nurses employed by the trust, using email communications and web-based meeting forums. Nurses who were interested in taking part were provided with a Qualtrics survey web link that contained a study information sheet and consent form. Upon providing consent, they were asked to provide demographic information and complete a prestudy survey.... The REsOluTioN program was hosted on the Totara learning management system (Version 12), via the Learning and Development information technology team at the participating trust. The web-based training was conducted over 4 weeks and covered weekly modules on (1) building hardiness and maintaining a positive outlook; (2) intellectual flexibility and emotional intelligence; (3) reflective and critical thinking; and (4) achieving life balance and enabling spirituality.
A blended synchronous and asynchronous learning approach was used that included (1) web-based 4×120 minutes large-group facilitated sessions on the weekly modules led by experienced senior nurses and other senior multidisciplinary health care staff; (2) 4×30 minute independent preparatory learning on the module topics prior to the large-group facilitated sessions; and (3) 8 small group mentoring sessions led by senior nurses and delivered between 30 and 60 minutes at flexible timings, twice weekly."

4a-iii) Information giving during recruitment

4b) CONSORT: Settings and locations where the data were collected

"It [the study] was conducted in a mental health and community National Health Service (NHS) trust in the South of England."

4b-i) Report if outcomes were (self-)assessed through online questionnaires

Participants completed online pre and post study surveys via the online Qualtrics platform.

"Nurses who were interested in taking part were provided with a Qualtrics survey web link that contained a study information sheet and consent form. Upon providing consent, they were asked to provide demographic information and complete a prestudy survey... After 6 weeks, participants from both arms were asked to complete a poststudy survey."

4b-ii) Report how institutional affiliations are displayed

5) CONSORT: Describe the interventions for each group with sufficient details to allow replication, including how and when they were actually administered

5-i) Mention names, credential, affiliations of the developers, sponsors, and owners

**5-ii) Describe the history/development process**		
**5-iii) Revisions and updating**		
**5-iv) Quality assurance methods**		
**5-v) Ensure replicability by publishing the source code, and/or providing screenshots/screen-capture video, and/or providing flowcharts of the algorithms used**		
**5-vi) Digital preservation**		
**5-vii) Access**		
"Nurses who were interested in taking part were provided with a Qualtrics survey web link that contained a study information sheet and consent form. Upon providing consent, they were asked to provide demographic information and complete a prestudy survey.<br>The REsOluTioN program was hosted on the Totara learning management system (Version 12), via the Learning and Development information technology team at the participating trust."		
**5-viii) Mode of delivery, features/functionalities/components of the intervention and comparator, and the theoretical framework**		
"The REsOluTioN program was hosted on the Totara learning management system (Version 12), via the Learning and Development information technology team at the participating trust. The web-based training was conducted over 4 weeks and covered weekly modules on (1) building hardiness and maintaining a positive outlook; (2) intellectual flexibility and emotional intelligence; (3) reflective and critical thinking; and (4) achieving life balance and enabling spirituality.<br>A blended synchronous and asynchronous learning approach was used that included (1) web-based 4×120 minutes large-group facilitated sessions on the weekly modules led by experienced senior nurses and other senior multidisciplinary health care staff; (2) 4×30 minute independent preparatory learning on the module topics prior to the large-group facilitated sessions; and (3) 8 small group mentoring sessions led by senior nurses and delivered between 30 and 60 minutes at flexible timings, twice weekly.<br>Both large-group facilitated sessions and mentor meetings were delivered via Teams (Microsoft Corporation). Learning materials in the form of PowerPoint presentations, instructional videos created by the study team, case examples, and peer-reviewed journal articles were used. The facilitated sessions also included group discussions and breakout activities. More about the content and delivery of REsOluTioN program is found elsewhere [37]. To gain a training completion certificate, participants randomized to the REsOluTioN program were expected to attend all the large-group facilitated sessions and at least one mentor meeting each week."		
**5-ix) Describe use parameters**		
**5-x) Clarify the level of human involvement**		
**5-xi) Report any prompts/reminders used**		
No prompts or reminders were used once participants had signed up to the trial.		
**5-xii) Describe any co-interventions (incl. training/support)**		
No additional training interventions were provided.		
**6a) CONSORT: Completely defined pre-specified primary and secondary outcome measures, including how and when they were assessed**		
"The following outcome data were collected:<br>(1) Participant Engagement: Data were collected on the number of nurses who expressed an interest in joining the trial and those randomized to assess recruitment success and participant engagement with the trial.<br>(2) Acceptability of the REsOluTioN program: Data were collected on how many participants were retained in the REsOluTioN program. Baseline Likert-style data were collected from all participants via a web-based prestudy survey to examine their understanding of resilience and the anticipated usefulness of the REsOluTioN tool in enhancing resilience levels, confidence, views on clinical practice, communication skills, and relationships with workplace colleagues. Six weeks later, participants were provided with this information again in a web-based poststudy survey to monitor any changes over time.<br>(3) Resilience and Psychological Well-being: The validated Brief Resilience Scale [40] and Warwick-Edinburgh Mental Wellbeing Scale [41] were used to measure changes in resilience, psychological health, and well-being over time between the REsOluTioN program and the waitlist control arms at 6 weeks."		
**6a-i) Online questionnaires: describe if they were validated for online use and apply CHERRIES items to describe how the questionnaires were designed/deployed**		
**6a-ii) Describe whether and how "use" (including intensity of use/dosage) was defined/measured/monitored**		
**6a-iii) Describe whether, how, and when qualitative feedback from participants was obtained**		
**6b) CONSORT: Any changes to trial outcomes after the trial commenced, with reasons**		
"It [the study] was conducted in a mental health and community National Health Service (NHS) trust in the South of England."		
**7a) CONSORT: How sample size was determined**		
**7a-i) Describe whether and how expected attrition was taken into account when calculating the sample size**		
**7b) CONSORT: When applicable, explanation of any interim analyses and stopping guidelines**		
"The following outcome data were collected:<br>(1) Participant Engagement: Data were collected on the number of nurses who expressed an interest in joining the trial and those randomized to assess recruitment success and participant engagement with the trial.<br>(2) Acceptability of the REsOluTioN program: Data were collected on how many participants were retained in the REsOluTioN program. Baseline Likert-style data were collected from all participants via a web-based prestudy survey to examine their understanding of resilience and the anticipated usefulness of the REsOluTioN tool in enhancing resilience levels, confidence, views on clinical practice, communication skills, and relationships with workplace colleagues. Six weeks later, participants were provided with this information again in a web-based poststudy survey to monitor any changes over time.<br>(3) Resilience and Psychological Well-being: The validated Brief Resilience Scale [40] and Warwick-Edinburgh Mental Wellbeing Scale [41] were used to measure changes in resilience, psychological health, and well-being over time between the REsOluTioN program and the waitlist control arms at 6 weeks."		
**8a) CONSORT: Method used to generate the random allocation sequence**		
"An independent team member who was not involved in the conduct of the trial, delivery of REsOluTioN program, or data analysis implemented the randomization and allocation. For randomization, we used a computer-generated random number sequence. For allocation concealment, we used sequentially numbered opaque-sealed envelopes that were opened only after entering the name of each participant on the envelope. A team member who was involved in data analysis was blinded to the group allocation. However, participants were not blinded to group allocation due to the nature of the training."		
**8b) CONSORT: Type of randomisation; details of any restriction (such as blocking and block size)**		
"The study was a 1:1 two-armed pilot randomized trial...An independent team member who was not involved in the conduct of the trial, delivery of REsOluTioN program, or data analysis implemented the randomization and allocation. For randomization, we used a computer-generated random number sequence. For allocation concealment, we used sequentially numbered opaque-sealed envelopes that were opened only after entering the name of each participant on the envelope. A team member who was involved in data analysis was blinded to the group allocation. However, participants were not blinded to group allocation due to the nature of the training."		
**9) CONSORT: Mechanism used to implement the random allocation sequence (such as sequentially numbered containers), describing any steps taken to conceal the sequence until interventions were assigned**		
""The study was a 1:1 two-armed pilot randomized trial...An independent team member who was not involved in the conduct of the trial, delivery of REsOluTioN program, or data analysis implemented the randomization and allocation. For randomization, we used a computer-generated random number sequence. For allocation concealment, we used sequentially numbered opaque-sealed envelopes that were opened only after entering the name of each participant on the envelope. A team member who was involved in data analysis was blinded to the group allocation. However, participants were not blinded to group allocation due to the nature of the training."		
**10) CONSORT: Who generated the random allocation sequence, who enrolled participants, and who assigned participants to interventions**		
"An independent team member who was not involved in the conduct of the trial, delivery of REsOluTioN program, or data analysis implemented the randomization and allocation...A team member who was involved in data analysis was blinded to the group allocation. However, participants were not blinded to group allocation due to the nature of the training."		
**11a) CONSORT: Blinding - If done, who was blinded after assignment to interventions (for example, participants, care providers, those assessing outcomes) and how**		
**11a-i) Specify who was blinded, and who wasn't**		
"A team member who was involved in data analysis was blinded to the group allocation. However, participants were not blinded to group allocation due to the nature of the training."		
**11a-ii) Discuss e.g., whether participants knew which intervention was the "intervention of interest" and which one was the "comparator"**		

11b) CONSORT: If relevant, description of the similarity of interventions

Not applicable as comparator group was wait list control.

12a) CONSORT: Statistical methods used to compare groups for primary and secondary outcomes

"Participants' demographic characteristics and acceptability outcomes (completers/non-completers) were descriptively analyzed. Depending on the normality of the data, resilience and psychological well-being measures were presented as means (SD) or medians (IQR). Intention-to-treat analysis was carried out to examine outcomes. Data from participants who withdrew from the study and who were lost to follow up (n=14, 13.1%) were imputed using the expectation maximization technique. We used ANOVA to evaluate any differences in resilience and psychological well-being between arms at 6 weeks. All analyses were undertaken in SPSS statistics software, version 22.0 (IBM Corp) [43], with a significance level set at 0.05."

12a-i) Imputation techniques to deal with attrition / missing values

"Data from participants who withdrew from the study and who were lost to follow up (n=14, 13.1%) were imputed using the expectation maximization technique. "

12b) CONSORT: Methods for additional analyses, such as subgroup analyses and adjusted analyses

No additional statistical analyses were undertaken.

RESULTS

13a) CONSORT: For each group, the numbers of participants who were randomly assigned, received intended treatment, and were analysed for the primary outcome

"Between August 2021 and May 2022, 134 nurses expressed an interest in participating in the study. Of 134, 108 completed the web-based consent process and prestudy survey. One participant was excluded on the basis of eligibility as he/she was a nursing student rather than an employed member of staff. Consented participants (n=107) were randomly assigned to the waitlist control (n=51) or REsOluTioN program (n=56). Nine participants withdrew from the REsOluTioN group prior to the intervention starting due to changing work commitments or annual leave requirements, which meant that they were unable to be allocated to a training cohort. At 6 weeks postenrollment, 93 participants had completed the poststudy survey, as 5 were lost to follow up and did not complete (2 in the waiting list group and 3 in the REsOluTioN group)."

13b) CONSORT: For each group, losses and exclusions after randomisation, together with reasons

This data is provided in the CONSORT flow diagram within the manuscript

13b-i) Attrition diagram

14a) CONSORT: Dates defining the periods of recruitment and follow-up

"We carried out a pilot randomized trial (1:1), conducted at a single site (mental health and community trust in South England) between August 2021 and May 2022."

14a-i) Indicate if critical "secular events" fell into the study period

14b) CONSORT: Why the trial ended or was stopped (early)

The trial was not ended/did not stop early.

15) CONSORT: A table showing baseline demographic and clinical characteristics for each group

CharacteristicsTotalREsOluTioNa groupWaitlist control group
Number of participants randomized (n)1075651
Age in years, mean (SD)43.78 (10.85)44.04 (10.72)43.49 (11.08)
Gender, n (%)
Male 12 (11.2)7 (12.5)5 (9.8)
Female 95 (88.8)49 (87.5)46 (90.2)
Ethnicity, n (%)
White 95 (88.8)52 (92.9)43 (84.3)
Asian 3 (2.8)0 (0.0)3 (5.9)
Black 7 (6.5)2 (3.6)5 (9.8)
Mixed 2 (1.9)2 (3.6)0 (0.0)
Work experience in years, mean (SD)15.75 (11.58)14.88 (11.28)16.71 (11.94)
Level of NHSb bands, n (%)
Band 45 (4.7)4 (7.1)1 (2.0)
Band 516 (15.0)7 (12.5)9 (17.6)
Band 647 (43.9)26 (46.4)21 (41.2)
Band 721 (19.6)11 (19.6)10 (19.6)
Band 8 (a,b,c) 18 (16.8)8 (16.8)10 (19.6)
Type of work setting, n (%)
Community and mental health services91 (85.0)46 (82.1)45 (88.2)
Forensics4 (3.7)3 (5.4)1 (2.0)
Corporate6 (5.6) 2 (3.6) 4 (7.8)
Learning disabilities6 (5.6)5 (8.9)1 (2.0)
Resilience, mean (SD)
Baseline3.02 (0.27)3.01 (0.25)3.04 (0.28)
6 weeks3.02 (0.26)3.02 (0.25)3.02 (0.26)
Psychological well-being, mean (SD)
Baseline 46.35 (7.52)46.73 (8.32) 45.92 (6.59)
6 weeks 47.56 (9.26)48.55 (9.64)46.47 (8.78)

15-i) Report demographics associated with digital divide issues

CharacteristicsTotalREsOluTioNa groupWaitlist control group
Number of participants randomized (n)1075651
Age in years, mean (SD)43.78 (10.85)44.04 (10.72)43.49 (11.08)
Gender, n (%)
Male 12 (11.2)7 (12.5)5 (9.8)
Female 95 (88.8)49 (87.5)46 (90.2)
Ethnicity, n (%)
White 95 (88.8)52 (92.9)43 (84.3)
Asian 3 (2.8)0 (0.0)3 (5.9)
Black 7 (6.5)2 (3.6)5 (9.8)
Mixed 2 (1.9)2 (3.6)0 (0.0)
Work experience in years, mean (SD)15.75 (11.58)14.88 (11.28)16.71 (11.94)
Level of NHSb bands, n (%)
Band 45 (4.7)4 (7.1)1 (2.0)
Band 516 (15.0)7 (12.5)9 (17.6)
Band 647 (43.9)26 (46.4)21 (41.2)
Band 721 (19.6)11 (19.6)10 (19.6)
Band 8 (a,b,c) 18 (16.8)8 (16.8)10 (19.6)
Type of work setting, n (%)
Community and mental health services91 (85.0)46 (82.1)45 (88.2)
Forensics4 (3.7)3 (5.4)1 (2.0)
Corporate6 (5.6) 2 (3.6) 4 (7.8)
Learning disabilities6 (5.6)5 (8.9)1 (2.0)
Resilience, mean (SD)
Baseline3.02 (0.27)3.01 (0.25)3.04 (0.28)
6 weeks3.02 (0.26)3.02 (0.25)3.02 (0.26)
Psychological well-being, mean (SD)
Baseline 46.35 (7.52)46.73 (8.32) 45.92 (6.59)
6 weeks 47.56 (9.26)48.55 (9.64)46.47 (8.78)

16a) CONSORT: For each group, number of participants (denominator) included in each analysis and whether the analysis was by original assigned groups

16-i) Report multiple "denominators" and provide definitions

"Baseline characteristics of trial participants are presented in Table 1. The mean age of participants was 44 years (SD=10.85); ages ranged between 24 and 69 years. Most participants were female (n=95, 88.8%), White (n=95, 88.8%), and working in the community setting (n=91, 85.0%). Participants were from a range of NHS clinical bands (band 4--8), with band 6 being the most common (n=47, 43.9%). A small number (n=5) of band 4 nursing associates were included, as they were experienced members of staff embedded within clinical teams during the pandemic, working alongside registered nurses in extended roles. The mean number of years of experience working in the nursing profession was 15.75 (SD=11.58)....Between August 2021 and May 2022, 134 nurses expressed an interest in participating in the study. Of 134, 108 completed the web-based consent process and prestudy survey. One participant was excluded on the basis of eligibility as he/she was a nursing student rather than an employed member of staff. Consented participants (n=107) were randomly assigned to the waitlist control (n=51) or REsOluTioN program (n=56). Nine participants withdrew from the REsOluTioN group prior to the intervention starting due to changing work commitments or annual leave requirements, which meant that they were unable to be allocated to a training cohort. At 6 weeks postenrollment, 93 participants had completed the poststudy survey, as 5 were lost to follow up and did not complete (2 in the waiting list group and 3 in the REsOluTioN group)."

16-ii) Primary analysis should be intent-to-treat

17a) CONSORT: For each primary and secondary outcome, results for each group, and the estimated effect size and its precision (such as 95% confidence interval)

"Participants randomized to the REsOluTioN program (n=56) did not differ significantly from participants randomized to the control group (n=51) with regard to age (t105=-0.26, P=.80), years working in the profession (t105=0.82, P=.42), gender (□21=0.2, P=.66), ethnicity (□21=1.96, P=.16), banding (□21=0.3, P=.57), nursing field (□21=0.8, P=.38), well-being (t105=□0.56, P=.58)-, or resilience (t105=0.58, P=.56) outcomes at baseline (Table 1). The baseline, prestudy survey indicated that all participants (n=107, 100%) recognized the importance of personal resilience in the workplace, with the majority rating it as extremely important (n=88, 82.2%)."

"Participants were allocated to waitlist control and REsOluTioN groups in 4 consecutive cohorts during the recruitment period. Cohort 1 had 14 people in the waitlist and 6 in the REsOluTioN group; cohort 2 had 2 and 4; cohort 3 had 13 and 11; and cohort 4 had 22 and 26 participants, respectively. A total of 150 mentoring sessions and 16 facilitated sessions took place across the 4 cohorts. No mentors or facilitators reported the length of the sessions, but the mentoring sessions lasted between 30 minutes to 1 hour depending upon the number of participants allocated to the mentor, while the large-group facilitated sessions lasted up to 2 hours. Thus, the sessions were acceptable in terms of delivery and duration. The participant retention rate at 6 weeks was 96% (44/47) in the REsOluTioN group; of these, 33 participants (75%) provided extra feedback on the REsOluTioN program via an additional survey.

Of the participants who completed the poststudy evaluation of the REsOluTioN program (n=33), the majority thought that both the web-based workplace resilience training (n=30, 90.9%) and mentoring (n=29, 87.9%) had been useful; a minority felt that their participation in the training program had not impacted on their experience or outlook toward clinical practice (n=7, 21.2%). Participants felt that participation in the web-based training had been important for improving their levels of resilience (n=24, 72.8%), self-confidence (n=24, 72.7%), belief in their ability to provide good patient care (n=25, 75.8%), relationships with work colleagues (n=24, 72.7%), and communication skills with colleagues (n=25, 75.8%).
Most participants who completed poststudy evaluation also found the content and sessions helpful (n=29, 87.9%), thought the training delivered an appropriate amount of information (n=27, 81.8%), and that the 4-week duration was about right for training of this type (n=23, 69.7%). The sessions on emotional intelligence and intellectual flexibility were rated most favorably, with 75.8% (n=25) indicating that they found it particularly helpful.
Analysis of free text responses from REsOluTioN program participants suggested that they enjoyed opportunities for learning and reflection offered by the training as well as the chance to build networks and interact with colleagues at all levels. Participants indicated that they enjoyed the networking, mentorship, and participatory sessions the most (n=16, 51.5%). However, ringfencing time to attend and engage with the sessions was the most reported challenge due to pressurized working environments. One participant highlighted the need for a cultural shift to support people to take more time for their own well-being. Participants also highlighted organization-wide issues and variability in the way sessions were run as detracting from their overall experience. Table 2 provides a summary of illustrative quotes."

"Mean resilience and psychological well-being scores at baseline and 6 weeks for both groups are presented in Table 1. There were very little pre-post differences in resilience scores across control group (mean □0.01, SD 0.35) and REsOluTioN group (mean 0.01, SD 0.27). The mean pre-post difference in psychological well-being scores was smaller in the control group (mean 0.55, SD 6.77) compared to the REsOluTioN group (mean 1.82, SD 6.53).
Two-way mixed ANOVAs revealed no statistically significant differences between groups and time (baseline and 6 weeks) on resilience scores (F1,105=0.20, P=.66, partial η2=0.002) and well-being scores (F1,105=0.97, P=.33, partial η2=0.09)."

17a-i) Presentation of process outcomes such as metrics of use and intensity of use

17b) CONSORT: For binary outcomes, presentation of both absolute and relative effect sizes is recommended

This information was not required for the study outcomes.

18) CONSORT: Results of any other analyses performed, including subgroup analyses and adjusted analyses, distinguishing pre-specified from exploratory

Not relevant to this study.

18-i) Subgroup analysis of comparing only users

19) CONSORT: All important harms or unintended effects in each group

Not relevant to this study.

19-i) Include privacy breaches, technical problems

19-ii) Include qualitative feedback from participants or observations from staff/researchers

DISCUSSION

20) CONSORT: Trial limitations, addressing sources of potential bias, imprecision, multiplicity of analyses

20-i) Typical limitations in ehealth trials

"To our knowledge, REsOluTioN was the first web-based training piloted on nurses working during the COVID-19 pandemic and has highlighted many benefits of providing such a resource to nurses working under highly pressurized conditions. In addition, the engagement and acceptability outcomes have been achieved as indicated by the high study recruitment and adherence rates and from the qualitative survey feedback. This confirms that it is possible to implement web-based resilience training programs for nurses within busy workplace environments with successful engagement from nurses.

Study limitations include the small sample size, and hence robust conclusions on psychological well-being and resilience outcomes cannot be made. In addition, this study was conducted at only one NHS trust; future studies should be conducted across a range of NHS health and social care settings to increase the generalizability of the findings. Finally, conducting a study during the COVID-19 pandemic may have influenced the findings, as nurses were working under even more pressurized conditions than usual [1,23,44], which may have influenced their responses due to heightened levels of stress and anxiety. Care must be taken to be aware of these issues when implementing similar resources across health and social care settings in the peri- and postpandemic era."

21) CONSORT: Generalisability (external validity, applicability) of the trial findings

21-i) Generalizability to other populations

21-ii) Discuss if there were elements in the RCT that would be different in a routine application setting

22) CONSORT: Interpretation consistent with results, balancing benefits and harms, and considering other relevant evidence

22-i) Restate study questions and summarize the answers suggested by the data, starting with primary outcomes and process outcomes (use)

"Our findings have shown that nurse participants engaged with REsOluTioN program, as evidenced by the high recruitment rate to the pilot study. The training was acceptable to nurses working in frontline clinical settings; this is demonstrated by the large number of participants who enrolled in and completed the study. While a small number of participants withdrew from the study or were lost to follow up, the majority of these withdrew prior to the training commencing due to changing work commitments, meaning that they were no longer able to join a cohort. Participant feedback demonstrated that most participants clearly valued the importance of protecting their resilience within the workplace. Most participants were receptive toward the REsOluTioN program and reported that they felt it was an important tool for helping to enhance their resilience, confidence, patient care provision, and relationships with work colleagues; this demonstrates the potential application of web-based resilience tools for this population group. Overall, the REsOluTioN program was well received and identified a desire from nurses for web-based resilience training tools to be implemented as a way of optimizing resilience, psychological health, communication practices, and the workplace environment. Though there were no significant differences between REsOluTioN and waitlist control groups at 6 weeks in terms of resilience and psychological well-being, these results must be considered alongside the small sample size, due to the pilot nature of the trial."

22-ii) Highlight unanswered new questions, suggest future research

Other information

23) CONSORT: Registration number and name of trial registry

"ClinicalTrials.gov NCT05074563" .

24) CONSORT: Where the full trial protocol can be accessed, if available

The full trial protocol has been published in JMIR Res Protoc:<br><br>Srikesavan, C, Davey, Z, Cipriani, A, Henshall, C (2022) Resilience Enhancement Online Training for Nurses (REsOluTioN): Protocol for a Pilot Randomised Controlled Trial. JMIR Res Protoc 2022;11(8):e37015 doi: 10.2196/37015 PMID: 35862692		
**25) CONSORT: Sources of funding and other support (such as supply of drugs), role of funders**		
"This study is funded by the Burdett Trust for Nursing (SB\ZA\101010662\633134)."		
**X26-i) Comment on ethics committee approval**		
**x26-ii) Outline informed consent procedures**		
**X26-iii) Safety and security procedures**		
**X27-i) State the relation of the study team towards the system being evaluated**		
